# 白蛋白结合型紫杉醇在非小细胞肺癌治疗中的研究进展

**DOI:** 10.3779/j.issn.1009-3419.2010.07.16

**Published:** 2010-07-20

**Authors:** 鹏 李, 启鸣 王, 慧娟 王, 智勇 马

**Affiliations:** 450003 郑州，河南省肿瘤医院内科 Department of Medical Oncology, Tumor Hospital of Henan Province, Zhengzhou 450003, China

众所周知，紫杉醇是非小细胞肺癌（non-small cell lung cancer, NSCLC）化疗中重要的常用化疗药物，其溶剂为聚氧乙烯蓖麻油/乙醇，这种溶剂型紫杉醇容易产生严重的过敏反应，从而加重骨髓抑制、神经毒性等，因此在临床安全方面其使用受到很大限制；另外，溶剂型紫杉醇抑制白蛋白介导的药物转运，组织利用度较低，缺乏量效关系，影响合用药物的疗效，严重影响治疗效果。近些年来，一种新型紫杉醇——白蛋白结合型紫杉醇（albumin-bound paclitaxel; nab-paclitaxel）脱颖而出，弥补了溶剂型紫杉醇的各种不利影响，显示出较好的疗效和安全性。一系列细胞学和动物学研究^[1, 2]^显示，白蛋白结合型紫杉醇的独特作用机制体现在抗肿瘤的多个环节，包括药物转运、药物吸收、药物利用等方面，有助于紫杉醇发挥最大的功效。目前白蛋白结合型紫杉醇已通过国家食品药品监督管理局（SFDA）审批在中国上市，批准其治疗联合化疗失败的转移性乳腺癌以及辅助化疗6个月内复发的乳腺癌。除此之外，国外研究者还进行了一些白蛋白结合型紫杉醇在卵巢癌、前列腺癌、头颈部鳞癌等中的临床研究^[3-5]^，均取得了不错的疗效以及安全性。2009年美国肿瘤学年会（American Society of Clinical Oncology, ASCO）报道了白蛋白结合型紫杉醇在黑色素瘤、胰腺癌中应用的一系列Ⅰ/Ⅱ期临床研究，结果令人欣喜，有望成为该两种化疗不敏感肿瘤的有效药物，并且提示富含半胱氨酸的酸性分泌蛋白（secreted protein acidic and rich in cysteine, SPARC）阳性的患者缓解率更高、无进展生存时间（progression-free survival, PFS）更长^[6, 7]^。目前，肺癌发病率及死亡率居高不下，国外学者对白蛋白结合型紫杉醇在NSCLC治疗中的应用进行了很多临床研究。本文将对白蛋白结合型紫杉醇在NSCLC中应用的最新临床研究做一综述。

## 1 白蛋白结合型紫杉醇单药治疗或联合卡铂治疗进展期NSCLC

Allerton等^[8]^进行了一项Ⅱ期临床研究，入组41例NSCLC患者，进行白蛋白结合型紫杉醇三种不同方案的爬坡研究，分别为每周方案（4周为1周期，每周治疗，共进行3周），第1天、第8天方案（每21天为1周期）和每21天方案（第1天给药），均联合固定的卡铂剂量（AUC=6）。研究发现，其主要毒副作用为骨髓抑制。三种方案的最大耐受量分别为100 mg/m^2^、125 mg/m^2^和300 mg/m^2^。其也对100 mg/m^2^白蛋白结合型紫杉醇联合卡铂方案与紫杉醇联合卡铂方案的疗效进行了比较，结果发现前者神经毒性发生率较低（0% *vs* 5%），但是血小板减少的发生率较高（18% *vs* 5%）。另外，Hawkins等^[9]^进行了一项Ⅰ/Ⅱ期临床研究，入组25例NSCLC患者，分别给予225 mg/m^2^、260 mg/m^2^、300 mg/m^2^、340 mg/m^2^白蛋白结合型紫杉醇联合卡铂（AUC=6），总有效率为27%。基于这些Ⅰ/Ⅱ期剂量爬坡以及安全性临床研究，一系列临床研究已逐渐开展。

### 1.1 白蛋白结合型紫杉醇一线治疗晚期NSCLC的疗效及安全性

标准的进展期NSCLC一线化疗大多为三周方案，Green等^[10]^进行了一项多中心、Ⅱ期临床研究，以探讨白蛋白结合型紫杉醇260 mg/m^2^一线治疗晚期NSCLC的疗效及安全性。2004年2月25日-2004年6月21日共入组43例患者，84%的患者伴有内脏转移，所有的患者ECOG评分为0分-1分。客观有效率（objective response rate, ORR）为16.3%（95%CI: 5.24-27.31），共有7例患者达到客观缓解，均为部分缓解；中位总生存（overall survival, OS）为11个月（95%CI: 9.5-16.2），中位疾病进展时间（time to progression, TTP）为6个月（95%CI: 9.5-16.2），预计1年生存率为45%。无4度不良反应发生，有2例患者出现3度外周神经毒性，其中1例患者因为毒副作用停止治疗，但总体上不良反应耐受良好。总之，在疾病进展时间和中位总生存期方面，白蛋白结合型紫杉醇与紫杉醇联合卡铂两药方案的疗效相似，并且毒副作用可以耐受，因此可以进行白蛋白结合型紫杉醇联合铂类的临床研究。

### 1.2 白蛋白结合型紫杉醇每周方案治疗Ⅳ期NSCLC的最大耐受量和单药有效率

在乳腺癌中，剂量强度和剂量密度的观念日益深入。为了进一步探索提高白蛋白结合型紫杉醇疗效的方案，一些临床研究在进展期NSCLC中比较了每周方案和三周方案的疗效。Rizvi等^[11]^进行了一项Ⅰ期/Ⅱ期临床研究，以确定白蛋白结合型紫杉醇每周方案治疗Ⅳ期NSCLC的最大耐受量（maximum tolerance dose, MTD）和单药有效率。该研究为开放、单组、Ⅰ期/Ⅱ期研究。患者接受NAB-紫杉醇30 min内静脉注射，第1天、第8天和第15天，每28天为1周期，并且不提前应用激素或抗组胺药物。每8周进行肿瘤的影像学评估。结果显示，患者可以很好地耐受100 mg/m^2^和125 mg/m^2^的剂量水平，并且无剂量限制性毒性（dose-limiting toxicity, DLT）发生。MTD低于150 mg/m^2^；3例患者中有2例出现DLT（3度感觉神经损伤和粒细胞缺乏性发热）。因此125 mg/m^2^被认为是白蛋白结合型紫杉醇每周方案的最大耐受量。在本研究的Ⅱ期临床阶段，共有40例患者接受125 mg/m^2^的治疗，有效率为30%（40例患者中有12例患者出现客观缓解；95%CI: 16-44），中位TTP时间为5个月（95%CI: 3-8），中位总生存期为11个月（95%CI: 7-未达到）。1年生存率为41%。其有效率与ECOG1594研究^[12]^中含铂双药方案相当。因此白蛋白结合型紫杉醇125 mg/m^2^，第1天、第8天、第15天用药的每周方案，耐受性好，单药治疗有效率很有前景。

### 1.3 2009年世界肺癌大会有关白蛋白结合型紫杉醇的报道

在2009年世界肺癌大会发表了两篇关于白蛋白结合型紫杉醇的文章摘要。第一项临床研究为非随机、开放的Ⅱ期临床研究，主要探讨白蛋白结合型紫杉醇每周方案和3周方案联合卡铂一线治疗进展期NSCLC的疗效^[13]^。入组患者为≥18岁、且均未经过治疗的Ⅲb/Ⅳ期NSCLC患者。白蛋白结合型紫杉醇爬坡剂量梯度分别7组，分别为三周方案组225 mg/m^2^、260 mg/m^2^、300 mg/m^2^、340 mg/m^2^；每周方案组：①每周1次，连用2周，休息1周，140 mg/m^2^；②每周1次，连用3周，不休息，100 mg/m^2^、125 mg/m^2^，所有研究亚组的患者均接受卡铂（AUC=6）第1天，每3周为1周期。共有175例患者入组，每组25例患者，各组间基线水平相似。每周方案的客观有效率更高，为47%，而三周方案为30%。在每周方案组中的中位PFS分别为5.6个月、6.2个月和6.4个月，总的中位PFS为6.1个月，中位OS为12.3（11.3-15）个月。在三周方案组中，中位PFS为5.9（4.8-6.9）个月，中位OS为12.2（8.3-14.6）个月。每周方案组和三周方案组的疾病控制率（disease control rate, DCR）（≥16周的疾病稳定或客观缓解）分别为56%和46%。总体来说，常见的3/4度非血液学毒副作用为外周神经炎（19%）和乏力（9%）。最常见的血液学3/4度毒副作用为中性粒细胞减少（60%）。在每组≥10%的毒副作用中，与三周方案相比，每周方案中外周神经炎、肌痛、关节痛和脱发的发生率较低。在接受100 mg/m^2^白蛋白结合型紫杉醇的患者中，3/4度中性粒细胞减少和血小板减少的发生率最低。因此，白蛋白紫杉醇联合卡铂治疗进展期NSCLC安全有效。基于白蛋白结合型紫杉醇每周方案的疗效和安全性更好，目前已经启动一项Ⅲ期、随机、多中心临床研究对比100 mg/m^2^白蛋白结合型紫杉醇第1天、第8天、第15天联合卡铂（AUC=6，三周一次）和200 mg/m^2^溶剂型紫杉醇第1天联合卡铂（AUC=6，三周一次）。

另外一项Ⅱ期临床研究对上一项临床研究进行回顾性分析^[14]^，该研究者认为病理类型不同，有效率也不同。对接受治疗患者中的166例患者，进行病理类型分析。组间的基线水平相似。与三周方案相比，对于腺癌的患者来说，纳米紫杉醇每周方案的疗效更好[ORR分别为59.4%、23.5%，*P*=0.003]。虽然差异无统计学意义，接受每周方案纳米紫杉醇的腺癌患者的PFS和OS均较每三周方案好。与接受每周方案的鳞癌患者相比，接受三周方案的鳞癌患者，PFS显著提高（*P*=0.014），同时OS延长，但在ORR方面无差异（表 1）。

**1:** 每周方案与三周方案在鳞癌和腺癌中的疗效比较 Comparison of therapeutic effects between weekly schedule and every-3-week schedule

Squamous cell carcinoma	Weekly schedule (*n*=41)	Every-3-week schedule (*n*=59)	*P*	Adenocarnoma	Weekly schedule (*n*=32)	Every-3-week schedule (*n*=34)	*P*
ORR, *n* (%)	16 (39)	21 (36)	0.727		19 (59)	8 (24)	0.003
PFS (month)	4.8	7.7	0.041		7.7	5.3	0.070
OS (month)	12.0	14.6	0.339		13.2	10.7	0.341

**2:** 含贝伐单抗不同化疗方案的对比 Comparison of different regimens with bevacizumab

Regimens	Maintenance with Bev	ORR (%)	OS (month)	PFS (month)
nab-Pax+Cb+Bev^[15]^	No	31%	16.8	9.8
Gem+C+Bev^[16]^	Yes	20.1%	-	6.7
Pax+Cb+Bev^[17]^	Yes	35%	12.3	6.2
Pem+Cb+Bev^[18]^	Yes	55%	14.1	7.8

该回顾性分析充分体现了个体化治疗。腺癌和鳞癌生物学行为不同，对药物治疗的反应也不尽相同，这项回顾性分析充分证明这一问题，并对临床实践具有重要意义，一定程度上提示：如果患者为肺鳞癌可以选择白蛋白结合型紫杉醇三周方案，而为肺腺癌则选择每周方案进行治疗，但尚需进一步的Ⅲ期临床研究验证。

## 2 白蛋白结合型紫杉醇/卡铂联合贝伐单抗一线治疗进展期非鳞癌NSCLC的Ⅱ期临床研究

卡铂/紫杉醇方案联合贝伐单抗是治疗进展期非鳞癌NSCLC的标准方案。白蛋白结合型紫杉醇解决了许多常规溶剂溶解的紫杉醇输注难题，并在疗效方面进一步提高。因此Reynolds等^[15]^评价了白蛋白结合型紫杉醇/卡铂联合贝伐单抗在治疗进展期非鳞癌NSCLC患者的疗效。2005年10月-2006年4月，共有50例Ⅲb期/Ⅳ期NSCLC患者入组；其中48例患者接受白蛋白结合型紫杉醇300 mg/m^2^、卡铂AUC=6、贝伐单抗15 mg/kg，每21天为1周期，直至疾病进展或出现不可耐受的毒副作用，最多进行4周期，可以根据患者具体情况及临床医生的建议慎重选择再进行2周期；不进行贝伐单抗维持治疗。入组人群中，56%的患者为女性，中位年龄为67（37-83）岁，PS评分0分（52%）或1分（48%），腺癌占86%，Ⅳ期患者占82%。接受评价的患者至少进行4周期。主要研究终点为客观有效率。结果显示：客观有效率为31%，稳定率为54%。无完全缓解出现。中位PFS为9.8个月（< 1个月-22.3个月），中位生存期为16.8个月。最常见的3度或4度治疗相关毒性为中性粒细胞减少（54%）和乏力（17%）。虽然本研究的客观有效率不高，仅为31%，但其稳定率高达54%，OS达到16.8个月，与AVAIL研究^[16]^（OR为30.4%）和ECOG4599研究^[17]^（OS为12.3个月，OR为35%）相比，其疗效已不错。另外，Patel等^[18]^实施的培美曲塞+卡铂+贝伐单抗一线治疗非鳞癌NSCLC、随后采用培美曲塞+贝伐单抗维持治疗的Ⅱ期临床研究结果显示，客观有效率为55%，中位无进展时间和中位总生存期分别为7.8个月和14.1个月。虽然两项临床研究的方案不同，入组患者情况亦相同，但结果均证实了白蛋白结合型紫杉醇优异的疗效。

## 3 白蛋白结合型紫杉醇/卡铂联合同步放疗治疗不可切除的Ⅲ期NSCLC的Ⅰ/Ⅱ期临床研究

在Ⅲ期NSCLC的同步放化疗方案中，紫杉醇联合卡铂同步放化疗的中位OS为14个月^[19]^。为了进一步评价白蛋白结合型紫杉醇/卡铂联合放疗的疗效，Keedy等^[20]^进行了一项Ⅰ/Ⅱ期临床研究，该研究白蛋白结合型紫杉醇的爬坡剂量初始为40 mg/m^2^，以20 mg/m^2^为单位增加剂量，联合卡铂（AUC=2），均为每周方案，共进行7周，同步放疗分割33次，然后进行2周期巩固化疗，方案为每周白蛋白结合型紫杉醇（100 mg/m^2^）/每周卡铂（AUC=6）。共有5例患者进行了2个剂量水平的白蛋白结合型紫杉醇治疗，分别为40 mg/m^2^、60 mg/m^2^。其中1例患者要求退出。直至目前，共有4例患者完成所有化疗，其中1例未减量。在同步治疗期间，2度以上的毒副作用分别为2度血小板减少（1例）、贫血（2例）、食管炎（1例）和乏力（2例），3度以上的毒副作用为缺氧（1例）。在巩固治疗阶段，2度毒副作用为贫血（1例），3度毒副作用为低血压（1例）、低磷血症（1例）。4例患者均可评价疗效，2例部分缓解，2例稳定。有2例患者分别于开始治疗后5个月和7个月出现进展，于开始治疗2例患者分别稳定8个月和10个月。因此，每周方案白蛋白结合型紫杉醇60 mg/m^2^联合卡铂同步放疗，安全有效，可以继续进行Ⅰ期临床研究，白蛋白结合型紫杉醇在同步化放疗中很有前景。

## 4 讨论

白蛋白结合型紫杉醇每周方案单药一线治疗Ⅳ期NSCLC，1年生存率达到41%，令人鼓舞，这依赖于其独特的抗肿瘤机制，能够在避免药物不良反应增加的基础上提高紫杉醇剂量，达到增加疗效但不增加毒副作用的效果。白蛋白结合型紫杉醇联合卡铂治疗NSCLC的总生存期突破1年，但是否存在鳞癌和腺癌、三周方案和每周方案等之间的差异还需进一步临床研究加以证实。在不进行贝伐单抗维持的情况下，白蛋白结合型紫杉醇/卡铂联合贝伐单抗可使患者无进展生存期达到9.8个月。一线治疗优势人群的IPASS研究^[21]^中，吉非替尼组和紫杉醇/卡铂组的无进展生存期分别为5.7个月和5.8个月，我们也期待白蛋白结合型紫杉醇的Ⅲ期随机对照研究结果。贝伐单抗联合化疗是一线治疗进展期NSCLC重要的靶向药物，众多的临床研究均显示贝伐单抗联合化疗可延长PFS和OS，其与白蛋白结合型紫杉醇的联合方案进一步验证了既往研究结果，并且PFS得到改善。SPARC是肿瘤在生长过程中分泌的，功能类似于白蛋白受体，可吸引粘附白蛋白；白蛋白结合型紫杉醇利用SPARC蛋白的特性，能够聚集于肿瘤细胞上，因此如果肿瘤组织SPARC蛋白阳性，白蛋白结合型紫杉醇的疗效更好，这一结果在乳腺癌、胰腺癌和黑色素瘤的临床研究中得到证实^[6, 7, 22]^，其在肺癌中的疗效尚需进一步临床研究证实。

总之，白蛋白结合型紫杉醇是进展期NSCLC治疗的新型药物，低毒高效，不需预处理，临床应用安全，耐受性好，具有独特的抗肿瘤机制；在单药治疗、联合治疗以及同步放化疗等方面均有令人鼓舞的临床疗效。有关白蛋白结合型紫杉醇的进一步Ⅲ期临床研究将为临床医生提供更多的证据支持。
